# Comparison on Photosynthesis and Antioxidant Defense Systems in Wheat with Different Ploidy Levels and Octoploid Triticale

**DOI:** 10.3390/ijms19103006

**Published:** 2018-10-02

**Authors:** Haotian Mao, Mengying Chen, Yanqiu Su, Nan Wu, Ming Yuan, Shu Yuan, Marian Brestic, Marek Zivcak, Huaiyu Zhang, Yanger Chen

**Affiliations:** 1 College of Life Sciences, Sichuan Agricultural University, Ya’an 625014, China; sicaumao@163.com (H.M.); sicauchenmy@163.com (M.C.); sicauwunan@163.com (N.W.); yuanming@sicau.edu.cn (M.Y.); 2 College of Life Sciences, Sichuan University, Chengdu 610061, China; snowdream215@163.com; 3 College of Resources Science and Technology, Sichuan Agricultural University, Chengdu 611130, China; anty9826@126.com; 4 Department of Plant Physiology, Slovak Agricultural University, 94976 Nitra, Slovakia; marian.brestic@uniag.sk (M.B.); marek.zivcak@uniag.sk (M.Z.)

**Keywords:** photosynthetic characteristics, antioxidative enzymes, wheat, polyploidy, photosystems, chlorophyll fluorescence

## Abstract

To investigate the evolutionary differences of wheat with different ploidy levels and octoploid Triticale, photosynthetic capacity, and antioxidant defenses system were compared within and between diploid, tetraploid and hexaploid wheat, and octoploid Triticale seedlings. The results showed that seed germination rate, chlorophyll content, and photochemical activity of photosystems, and the activities of antioxidative enzymes in hexaploid wheat and octoploid Triticale were significantly higher than in diploid and tetraploid wheat. Compared to other two wheat species and octoploid Triticale, hexaploid wheat presented lower levels of reactive oxygen species (ROS). Furthermore, we found that the levels of photosystem II reaction center protein D1, light-harvesting complex II b4 (CP29), and D subunit of photosystem I (PsaD) in diploid wheat were significantly lower compared with hexaploid wheat and octoploid Triticale. Taken together, we concluded that hexaploid wheat and octoploid Triticale have higher photosynthetic capacities and better antioxidant systems. These findings indicate that different ploidy levels of chromosome probably play an important regulatory role in photosystems and antioxidative systems of plants.

## 1. Introduction

Wheat (*Triticum aestivum* L.) is one of the most important cereal crops and staple foods in the world. In the last 30 years, a 1.2% increase in wheat global average annual yield/unit area has been found because of the combination of improved cultivars and advanced agronomic practices [1,2]. As a human-made crop, Octoploid Triticale (2n = 8x = 56, AABBDDRR) is the first Triticale produced and a new potential crop. It is regarded to combine the quality of common wheat and the resistance to abiotic stresses of rye. Wheat is regarded to be an important source of carbohydrates and has many vitamins minerals, and proteins [1]. However, the detailed photosynthetic efficiency and the antioxidative capacity are unknown within and between wheat with different ploidy levels and octoploid Triticale.

It is well known that the first domestication of wheat occurred about 10,000 years ago [3]. The long history of wheat domestication has shown the development of polyploidy and the adaptation to different climatic regimes [4]. Moreover, difference in traits and environment tolerance were also shown in plant materials representing different stages of wheat improvement [5]. Based on the number of chromosomes, *Triticum* genus is classified into three main species: diploid (2n = 2x = 14), tetraploid (2n = 4x = 28), and hexaploid (2n = 6x = 42) [6]. As shown in Figure S1, the tetraploid wheat (AABB) derived from natural hybridization between the diploid grass *Triticum* (*Aegilops*) species and the diploid wheat (*Triticum urartu*). Hexaploid wheat (AABBDD) is one of the most famous allopolyploid species and produced through the human-made method (Figure S1). However, octoploid Triticale (AABBDDRR) was obtained from the crossing of wheat and rye [7].

Many studies have showed that some morphological and physiological traits in the domestication and selection of wheat are mainly influenced by nuclear genomes [2,8,9,10]. Previous studies indicated that the D genome was involved in flag leaf area, gas exchange, chlorophyll content, and the net photosynthesis rate [11,12]. A genome of wheat might contain the genes that regulate the growth of roots, water use efficiency, and net photosynthesis rate [13,14]. R genome of wheat probably contains the genes that control the high water-utilizing efficiency and stomatal conductance [15]. Furthermore, B genome of wheat was probably involved in the stomatal resistance and photosynthetic rate [16]. Several studies found that transpiration rate and photosynthetic rate decreased occasionally with the increase in the ploidy levels of wheat during the development process [17,18]. Therefore, the different genomes and the ploidy levels of chromosomes seem to play different roles in morphological, physioloigical and biochemical characteristics of wheat. Although many researchers have reported that the effects of genome and different polidy on grain size, seedling growth, and photosynthesis of wheat [8,19,20], much less attention has been paid to the detailed photosynthetic characteristics and the antioxidative capacity among the three wheat species with different ploidy levels and octoploid Triticale.

Here, the photosynthesis and antioxidant system of wheat species differing in ploidy level and octoploid Triticale were investigated by comparing the difference in seed germination, plant growth, photosynthetic efficiency, gas exchange parameters, the activities of antioxidative enzymes, and the amounts of photosynthetic proteins. The goal of the present research was to evaluate the influence of different genomes and ploidy levels of chromosome on photosynthetic performance and the antioxidant system. Our results suggest that different ploidy levels of chromosomes are involved in the antioxidant defense system and the photosynthetic capacity in wheat species and octoploid Triticale.

## 2. Results

### 2.1. High Ploidy Levels Are Associated with Better Growth

**Figure 1:**
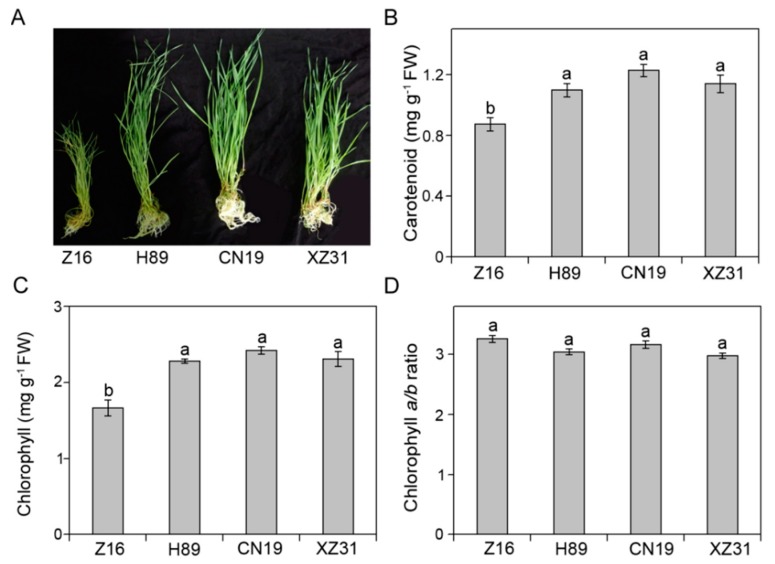
Phenotype (**A**), carotenoid (**B**), chlorophyll content (**C**), and chlorophyll *a*/*b* ratio (**D**) in Z16, H89, CN19, and XZ31. Values are means ± SD from four independent biological replicates (*n* = 4). Different letters indicate significant differences among Z16, H89, CN19, and XZ31 (*p* < 0.05) following Duncan’s multiplication range test. Z16, H89, CN19, and XZ31 present accessions of *Triticum monococcum* (W2n), *Triticum dicoccum* (W4n), *Triticum aestivum* (W6n), and octaploid Triticale (T8n), respectively.

To study the effects of different ploidy levels of chromosomes on the growth and yield, several growth and yield indexes were compared among the three wheat species with different ploidy levels and octoploid Triticale. As shown in Figure 1A, the phenotypes were different among the three wheat species with different ploidy levels and octoploid Triticale. Among these plants, Z16 (*Triticum monococcum*) presented the slowest growth rates and the smallest size, suggesting that wheat species with lower ploidy levels accumulate less biomass. Furthermore, plant height, root length, leaf area, and total length of Z16 were significantly lower than those in H89 (*Triticum dicoccum*), CN19 (*Triticum aestivum*), and XZ31 (octaploid Triticale) (Table 1). Compared with Z16, H89, and XZ31, CN19 showed the highest seed germination. In addition, we also found that CN19 presented the highest 1000-grain weight (52.8 g) compared to Z16, H89, and XZ31 (Table 1). These results indicated that CN19 and XZ31 had good growth, suggesting that high ploidy levels of chromosomes are probably associated with better growth in wheat species. 

### 2.2. High Ploidy Levels Are Associated with Higher Pigment Content, Proline Content, Total Protein, Soluble Sugar, But Lower Stomatal Density

To find out the reasons about the differences in the growth and yield the differences among the three wheat species with different ploidy levels and octoploid Triticale, several physiological parameters were further investigated. The differences in pigment contents among Z16, H89, CN19, and XZ31 were shown in Figure 1B–D. Chlorophyll (Chl) and carotenoid contents showed no significant differences among H89, CN19, and XZ31 (Figure 1B,C). However, Z16 presented the lowest contents of Chl and carotenoid. Furthermore, there was no significant difference in Chl *a*/*b* among Z16, H89, CN19, and XZ31 (Figure 1D). Chl showed a strong positive correlation with 1000-grain weight (Table S1). In addition, we also found that the contents of soluble sugar, soluble protein, and proline in Z16 were significantly lower than those in H89, CN19, and XZ31 (Table 2). Although there were no significant differences in the contents total protein and proline among H89, CN19, and XZ31, soluble sugar content in XZ was the highest compared with Z16, H89, and CN19. Furthermore, the stomatal properties in the second leaf were analyzed among Z16, H89, CN19, and XZ31 (Figure S2 and Table 2). We found that the number and size of stomata were different among Z16, H89, CN19, and XZ31 (Figure S2). In addition, Z16 and XZ31 presented the lowest and highest perimeter and area of single stomata (Table 2). In contrast, the order of stomatal density was Z16 > H89 > CN19 > XZ31.

### 2.3. High Ploidy Levels Are Associated with Higher Photosynthetic Capacities

**Figure 2:**
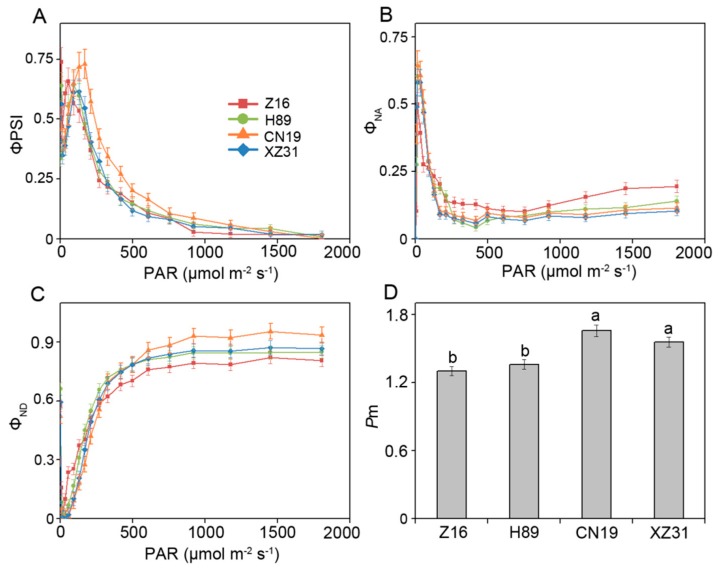
The values of parameters derived from P700 absorbance in leaves of Z16, H89, CN19, and XZ31. ΦPSI (**A**), effective quantum yield of PSI; Φ_NA_ (**B**), quantum yield of non-photochemical energy dissipation of PSI reaction centers due to acceptor side limitation; Φ_ND_ (**C**), quantum yield of non-photochemical energy dissipation in PSI reaction centers due to donor-side limitation (**C**); *P*m (**D**), maximal P700 signal. Values are means ± SD from four independent biological replicates (*n* = 4). Different letters indicate significant differences among Z16, H89, CN19, and XZ31 (*p* < 0.05) following Duncan’s multiplication range test. Z16, H89, CN19, and XZ31 present accessions of *Triticum monococcum* (W2n), *Triticum dicoccum* (W4n), *Triticum aestivum* (W6n), and octaploid Triticale (T8n), respectively.

The maximal P700 signal (*P*m) is considered to be interpreted as the maximum photo-oxidizable PSI content [21]. As shown in Figure 2, PSI photochemistry showed different changes among Z16, H89, CN19, and XZ31. PSI quantum yield (ΦPSI) in CN19 was higher than that of Z16, H89, and CN19 in low and moderate light intensities. In CN19 and XZ31, the quantum yield of non-photochemical energy dissipation of PSI reaction centers due to an acceptor side limitation (Φ_NA_) efficiently decreased in moderate and high light intensities, whereas the relatively high level of Φ_NA_ was observed in Z16, suggesting possible over reduction of the PSI acceptor side. Furthermore, the quantum yield of non-photochemical energy dissipation in PSI reaction centers due to donor-side limitation (Φ_ND_) retained higher level in CN19 as compared with Z16, H89, and XZ31 in moderate and high light intensities, indicating higher oxidation status of P700 in CN19. In addition, *P*m was significantly lower in Z16 and H89 as compared with CN19 and XZ31 (Figure 2D). Pearson correlation analysis suggested that *P*m and ΦPSI had better correlations with 1000-grain weight (Table S1)**.**

**Figure 3:**
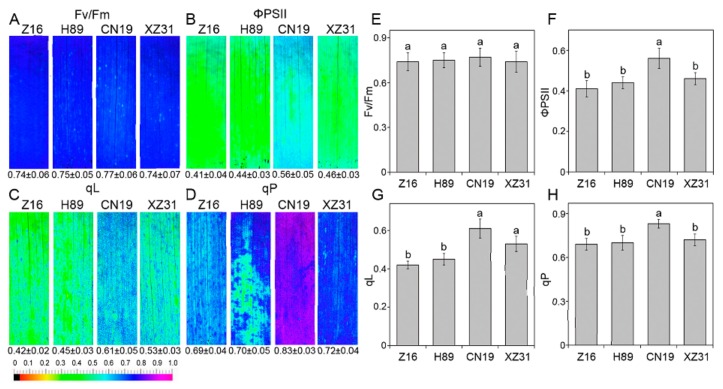
Chlorophyll fluorescence parameters (*Fv*/*Fm* (**A**,**E**), maximum efficiency of PSII photochemistry; ΦPSII (**B**,**F**), quantum yield of PSII electron transport; qL (**C**,**G**), fraction of PSII centers that are open; qP (**D**,**H**), photochemical quenching coefficient) in Z16, H89, CN19, and XZ31. Representative fluorescence images with quantitative data (±standard deviation) are presented on the left panel. Quantitative values (±SD) from four independent biological replicates are shown on the right panel. Different letters are significant differences at *p* < 0.05 level (Duncan’s multiple range test). Z16, H89, CN19, and XZ31 present accessions of *Triticum monococcum* (W2n), *Triticum dicoccum* (W4n), *Triticum aestivum* (W6n), and octaploid Triticale (T8n), respectively.

Next, PSII photochemistry among wheat with different ploidy levels and octoploid Triticale was determined by a modulated imaging fluorometer. The value and color of the maximum efficiency of PSII photochemistry (*Fv*/*Fm*) among Z16, H89, CN19, and XZ31 showed no significant differences (Figure 3A,E). However, we found that the values of the quantum yield of PSII electron transport (ΦPSII) (Figure 3B,F) and the photochemical quenching (qP) (Figure 3D,H) were obviously higher in CN19 compared to Z16, H89, and XZ31. Compared to Z16 and H89, CN19, and XZ31 presented the higher levels of the fraction of PSII centers in open states (qL) (Figure 3C,G). We also found that PSII photochemistry was strongly correlated with 1000-grain weight in these plants (Table S1).

**Figure 4:**
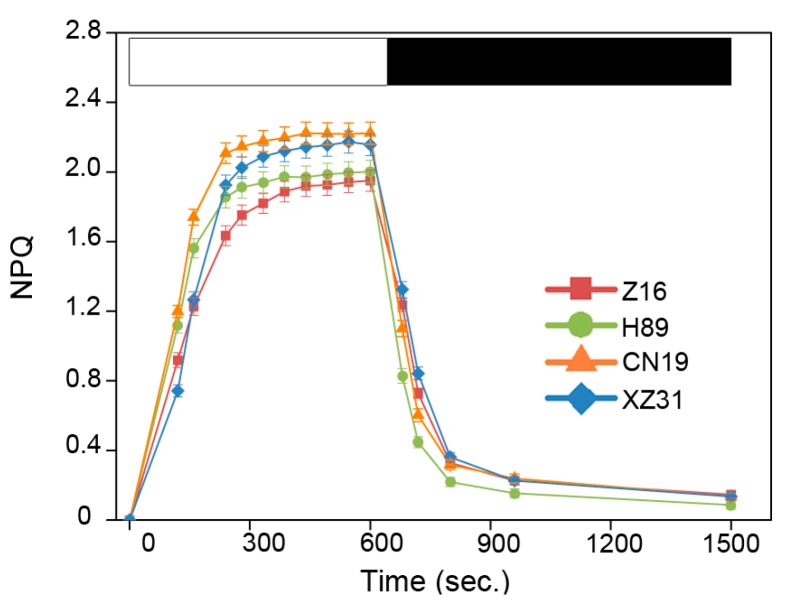
Non-photochemical quenching (NPQ) kinetics of Z16, H89, CN19, and XZ31 seedlings illuminated with 1000 µmol∙photons∙m^−2^∙s^−1^ for 10 min with a 15 min period darkness, as indicated by the white and black bars. Data represent an average of four independent measurements and are expressed as mean ± SD. Z16, H89, CN19, and XZ31 present accessions of *Triticum monococcum* (W2n), *Triticum dicoccum* (W4n), *Triticum aestivum* (W6n), and octaploid Triticale (T8n), respectively.

The non-photochemical quenching (NPQ) is the key photoprotection process in PSII. Excess light energy is harmlessly dissipated as heat by NPQ. NPQ values of wheat with different ploidy levels and octoploid Triticale were investigated in the present study (Figure 4). We found that CN19 and XZ31 had similar NPQ kinetics. Compared to CN19, induction of NPQ in Z16 and H89 was slower and reached a lower amplitude (1.8 at *t* = 10 min). Moreover, dark recovery was nearly the same among CN19, Z16, and XZ31. Except for the H89, the NPQ of CN19, Z16 and XZ31 relaxed to the same value in the dark. State transitions are thought to be good indicators of the energy conserving response [22]. We also measured the capacity of state I to state II transition among Z16, H89, CN19 and XZ31. As shown in Figure S3, there was no obviously difference among Z16, H89, CN19 and XZ31. Only a transitory increase of fluorescence in CN19 and XZ31 was observed when the first far-red light was on.

**Figure 5:**
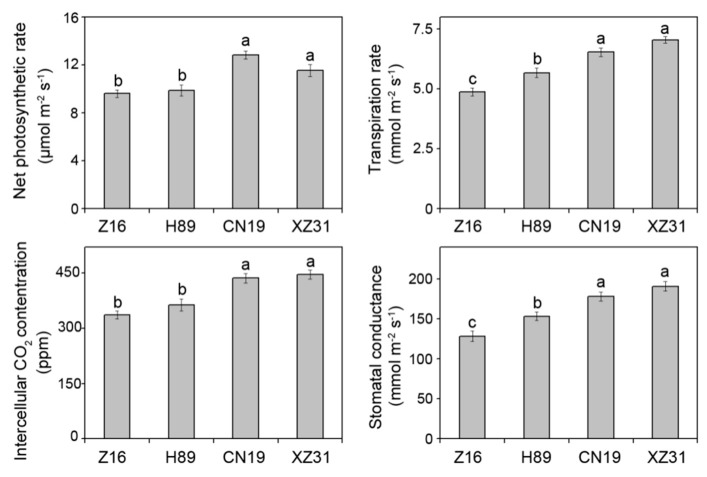
Net photosynthetic rate (*P*n), transpiration rate, intercellular CO_2_ concentration, and stomatal conductance of Z16, H89, CN19, and XZ31. Values are means ± SD from four independent biological replicates (*n* = 4). Different letters are significant differences at *p* < 0.05 level (Duncan’s multiple range test). Z16, H89, CN19, and XZ31 present accessions of *Triticum monococcum* (W2n), *Triticum dicoccum* (W4n), *Triticum aestivum* (W6n), and octaploid Triticale (T8n), respectively.

To further examine the differences in the photosynthetic capacities among wheat with different ploidy levels and octoploid Triticale, the net photosynthetic rate (*P*n), transpiration rate (Tr), intercellular CO_2_ concentration (Ci), and stomatal conductance (Gs) were measured (Figure 5). There were no significant differences in gas exchange parameters between CN19 and XZ31. Compared to CN19, Z16 and H89 presented the significant decrease in gas exchange parameters, especially in Z16. These results further demonstrated that CN19 and XZ31 have higher photosynthetic capacities.

### 2.4. High Ploidy Levels Are Associated with Lower ROS Accumulation

**Figure 6:**
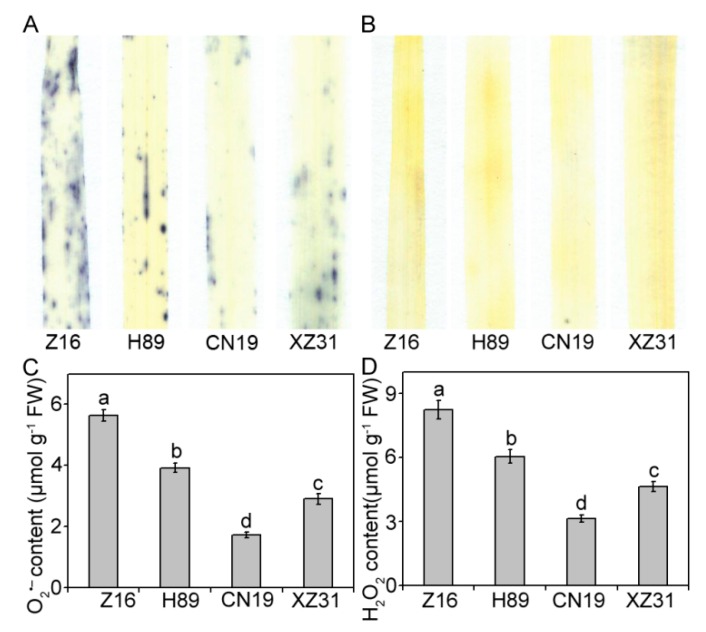
Measurement of reactive oxygen species (ROS) in Z16, H89, CN19, and XZ31. Histochemical analysis for superoxide anion radicals (O_2_^•–^) and hydrogen peroxide (H_2_O_2_) by nitro blue tetrazolium (NBT) (**A**) and 3,3-diaminobenzidine (DAB) (**B**) staining, respectively. Then, superoxide anion radicals (O_2_^•–^) production rate (**C**) and hydrogen peroxide (H_2_O_2_) content (**D**) were determined. Values are expressed as the means ± SD (*n* = 4). Different letters indicate significant differences at *p* < 0.05 level (Duncan’s multiple range test). Z16, H89, CN19, and XZ31 present accessions of *Triticum monococcum* (W2n), *Triticum dicoccum* (W4n), *Triticum aestivum* (W6n), and octaploid Triticale (T8n), respectively.

To investigate the differences in ROS production among wheat with different ploidy levels and octoploid Triticale, the levels of the two major ROS species, H_2_O_2_ and O_2_^•–^, were analyzed. Analysis of histochemical staining from the leaves using NBT and DAB showed that slight staining was observed in CN19 and XZ31 leaves (Figure 6A,B). However, Z16 presented the deep blue and yellow, suggesting high levels of O_2_^•–^ and H_2_O_2_, respectively. To confirm these results, the contents of O_2_^•–^ and H_2_O_2_ were measured. The results were almost identical to those observed by histochemical staining (Figure 6C,D). Compared to Z16, CN19 presented the lowest contents of H_2_O_2_ and O_2_^•–^ (52.1% and 74.2%, respectively).

### 2.5. High Ploidy Levels Are Associated with Higher Enzymatic and Non-Enzymatic Antioxidant Activities

**Figure 7:**
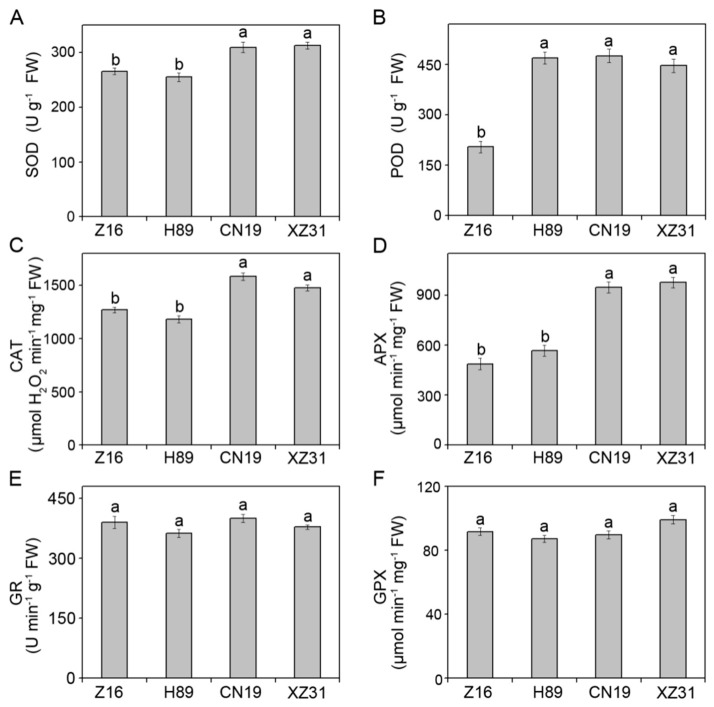
The activities of SOD, superoxide dismutase (**A**); POD, peroxidase (**B**); CAT, catalase; (**C**); APX, ascorbate peroxidase (**D**); GR, glutathione reductase (**E**); and GPX, glutathione peroxidase (**F**) in Z16, H89, CN19, and XZ31. Values are expressed as the means ± SD (*n* = 4). Different letters indicate significant differences at *p* < 0.05 level (Duncan’s multiple range test). Z16, H89, CN19, and XZ31 present accessions of *Triticum monococcum* (W2n), *Triticum dicoccum* (W4n), *Triticum aestivum* (W6n), and octaploid Triticale (T8n), respectively.

The differences observed among Z16, H89, CN19 and XZ31 regarding ROS accumulation led us to further investigate their antioxidant defense systems. Compared to CN19, XZ31 displayed similar activities of six antioxidant enzymes. Exception of peroxidase (POD), Z16 and H89 also showed the similar antioxidant enzyme activities. Compared to CN19, the activities of superoxide dismutase (SOD), POD, catalase (CAT) and ascorbate peroxidase (APX) in Z16 significantly decreased by 14.2%, 65.1%, 14.5%, and 50.2% (Figure 7A–D), respectively. However, there was no significant difference in activities of glutathione reductase (GR) and glutathione peroxidase (GPX) among Z16, H89, CN19 and XZ31 (Figure 7E,F). In addition, we also examined the contents of reduced ascorbic acid (AsA) and reduced glutathione (GSH), which are important antioxidant molecules. Although dehydroascorbate (DHA) and oxidized glutathione (GSSG) concentrations showed no obvious differences among Z16, H89, CN19 and XZ31, CN19 presented the highest concentrations of AsA and DHA compared to the other three plants (Figure S4). These results indicated that CN19 has a more efficient antioxidant defense system.

### 2.6. Ploidy Levels Are Not Associated with Thylakoid Membrane Protein Contents

**Figure 8:**
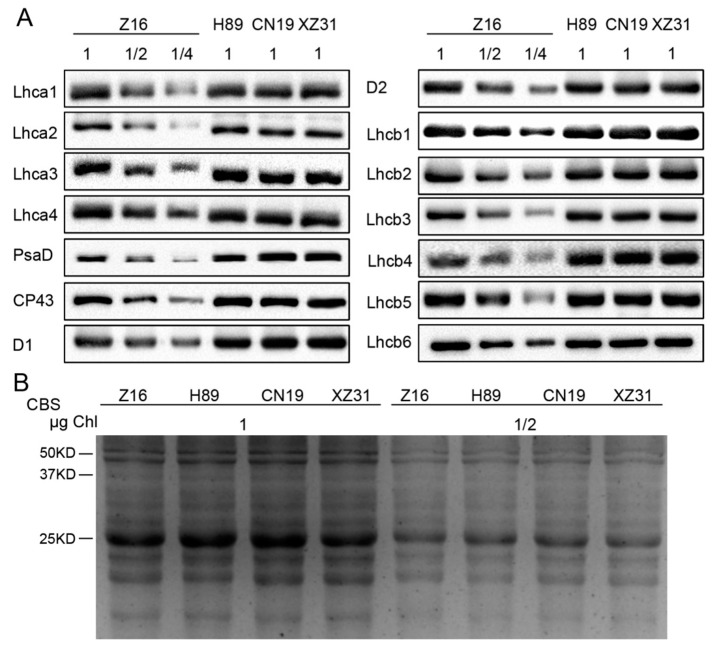
Immunoblot analyses of thylakoid proteins obtained from Z16, H89, CN19, and XZ31. Immunoblotting of thylakoid proteins were done using specific antibodies against representative photosystem I (PSI) (Lhca1, Lhca2, Lhca3, Lhca4, PsaD) and photosystem II (PSII) proteins (D1, D2, CP43, Lhcb1, Lhcb2, Lhcb3, Lhcb4, Lhcb5, Lhcb6) (**A**). Proteins corresponding to 0.25, 0.5, and 1 µg Chl (1/4, 1/2, and 1, respectively) were loaded. The SDS-PAGE results after Coomassie blue staining (CBS) were presented in the bottom panel (**B**). Loading was according to equal amount of total Chl (1 or 0.5 µg of Chl). Z16, H89, CN19, and XZ31 present accessions of *Triticum monococcum* (W2n), *Triticum dicoccum* (W4n), *Triticum aestivum* (W6n), and octaploid Triticale (T8n), respectively.

The differences in photosynthetic capacity are usually involved in the levels of thylakoid membrane proteins in plants. Therefore, the differences in the levels of PSI and PSII proteins were further analyzed by immunoblotting among Z16, H89, CN19 and XZ31 (Figure 8). Except for D1, PsaD and Lhcb4, almost no detectable changes in the amount of almost all the analyzed thylakoid membrane proteins were observed among Z16, H89, CN19, and XZ31 (Figure 8A). Interestingly, the levels of D1, PsaD, and Lhcb4 proteins showed significant decreases in Z16 compared to H89, CN19 and XZ31 (Figure 8A and Figure S5).

## 3. Discussion

It is well known that plant growth and photosynthetic characteristics have obvious differences in the process of wheat evolution [9,14,19]. Increased heterozygosity with increasing ploidy level may be an important one of their ecological and evolutionary successes relative to their diploid ancestors [23]. Many studies have indicated that domestication and ploidy level had significant effects on the morphological, physiological, biochemical, yield and yield components of wheat [2,10]. In the present study, we investigated the differences in the photosynthetic characteristics and antioxidant systems among wheat with different ploidy levels and octoploid Triticale.

It has been known that the photosynthetic pigments function as an important element of photosynthesis and play an important role in the absorption and conversion of light [1]. A previous study indicated that the reduction in chlorophyll content might be the reason for the low photosynthetic ability in hexaploidy [9]. Our results showed that the pigment contents in diploid wheat is significantly lower than others, indicating that high ploidy levels may be associated with higher photosynthetic pigment levels and higher photosynthetic capacities. The significant correlation between Chl and 1000-grain weight also indicated that hexaploid wheat and octoploid Triticale have high photosynthesis. Polyploids are associated with phenotypic plasticity, which may increase their range of ecological tolerance [24,25]. Our results showed that plant height and root length in hexaploid wheat and octoploid Triticale were significantly higher than those in diploid and tetraploid wheat (Figure 1 and Table 1). All these results indicated that the hexaploid wheat and octoploid Triticale had stronger morph-physiological adaptation.

It is well known that soluble sugar and proline are the two most important organic solutes and increase in response to environmental stresses in plants [26]. Many studies proposed that proline accumulation can be used to evaluate drought tolerance in cereals [27,28]. A recent study showed that soluble sugar concentration increased with the ploidy level under drought stress [2]. In the present experiment, we found that the levels of proline and soluble sugar in hexaploid wheat and octoploid Triticale were high. These findings may suggest that hexaploid wheat and octoploid Triticale have a better growth under sub-optimal growth conditions.

Although PSI is usually resistant to photoinhibition, the damage of PSI may be a more serious problem than PSII photoinhibition due to the slow recovery of PSI [29]. *P*m is the maximum P700 signal measured at saturation light pulse in a dark-adapted state [21]. In the present experiment, a significant decrease in photo-oxidizable PSI (*P*m) was observed in Z16, probably due to a result of a permanently increased reduction of PSI acceptor side (indicated by Φ_NA_ parameter). A previous report indicated that PSI inactivation was involved in the over reduction of PSI acceptor side (high value of Φ_NA_) [30]. Consistent with this finding, our results showed that CN19 and XZ31 presented low Φ_NA_ value, suggesting higher PSI activities in tetraploid wheat and octoploid Triticale. In addition, PSI photoinhibition might be alleviated when the photooxidation rate of P700 (Φ_ND_) in PSI exceeds the reduction rate of P700 [31]. Here, we found that the high value of Φ_ND_ in hexaploid wheat, suggesting that hexaploid wheat has a well capacity in alleviating the over-reduction of the PSI acceptor side.

Chlorophyll fluorescence (e.g., *Fv*/*Fm*, NPQ, qP and ΦPSII) has been considered to be non-invasive tool for the detection of plant photosynthetic performance under stressful and non-stressful conditions [32,33]. In the present study, the hexaploid wheat presented significantly high levels of qP, qL and ΦPSII (Figure 3). The high level of NPQ kinetics indicated that hexaploid wheat increases the efficiency of photochemical reactions of photosynthesis by dissipates effectively excess light energy [34]. In addition, hexaploid wheat showed high values for qL and qP, suggesting a greater fraction of open reaction centers and a greater fraction of maximum PSII efficiency, respectively [35]. Correlation analysis indicated that all chlorophyll fluorescence parameters were significant with 1000-grain weight (Table S1). The findings probably suggested that hexaploid wheat has a high photochemical efficiency. State transitions, the mechanisms by which photosystems balance their complements of LHCII between PSI and PSII depending on the reduction status of plastoquinone, are a good indicator in the process of energy dissipation [22]. In our study, state transitions did not present a significant difference among wheat with different ploidy levels and octoploid Triticale, probably suggesting that LHCII migration is similar among different plant species and octoploid Triticale. These results demonstrated that hexaploid wheat has a higher photosynthetic capacity compared to diploid and tetraploid wheat and octoploid Triticale. In addition, our results indicated that chlorophyll fluorescence was significantly correlated with crop yield especially, suggesting that photosynthetic parameters were probably good markers in the selection of plant species [1].

It has been known that photosynthesis can be affected via stomatal opening in plants. A previous study indicated that the stomatal length, width, perimeter, and area increased with the increase in ploidy levels [9]. Our results demonstrated that hexaploid wheat and octoploid Triticale had large stomata and low stomatal density (Table 2). However, a previous study showed that diploidy had higher *P*n and chlorophyll content than tetraploidy and hexaploidy [9]. Our results were not consistent with their findings. These differences may be attributed into different wheat species. A high value of *P*n in hexaploid wheat may be associated with the high crop biomass yield potential [11]. In addition, our correlation analysis showed that 1000-grain weight was significantly correlated with *P*n, probably suggesting hexaploid wheat has a high photosynthetic efficiency. A previous study indicated that stomatal conductance was a good screening technique for stressful tolerance in plants [36]. In our experiment, four gas exchange parameters (*P*n, stomatal conductance, transpiration rate, and intercellular CO_2_ concentration) in CN19 and XZ31 were higher than Z16 and H89. A similar result was also observed in wheat with different ploidy levels under drought stress [2]. Therefore, our findings suggested that hexaploid wheat and octoploid Triticale have higher photosynthesis than diploid and tetraploid wheat.

It has been well known that ROS plays a dual role in plants as important signal molecules or as toxic products of metabolism in cells, which may cause oxidative stress and photoinhibition [37]. This study showed that CN19 and XZ31 accumulated lower concentrations of ROS compared to Z16 and H89. Low ROS levels are thought to act as signal transduction molecules initiating several protective resistances [38]. Here, we proposed that hexaploid wheat and octoploid Triticale probably have a more effective ROS-scavenging system.

A complex array of enzymatic and non-enzymatic antioxidant defense systems are developed to maintain redox homeostasis in plants [39,40]. Oxidative damage can be eliminated or alleviated by the coordinated action of antioxidant enzymes and antioxidants. In our experiment, the activities of four antioxidant enzymes (SOD, POD, CAT, and APX) were significantly high in CN19 and XZ31, while markedly low in Z16 (Figure 7), suggesting that hexaploid wheat and octoploid Triticale have a good antioxidant enzymatic system. Previous studies have indicated that the AsA-GSH cycle is another important protection system against ROS damage to different cells [39]. Our results showed that high concentrations of AsA and GSH in hexaploid wheat and octoploid Triticale. Therefore, these results indicated that hexaploid wheat and octoploid Triticale have more effective enzymatic and non-enzymatic antioxidant systems, which may reduce oxidative damages resulted from excessive ROS production. In general, high ploidy levels are associated with higher enzymatic and non-enzymatic antioxidant activities, but low levels of ROS.

Our recent study showed that the levels of photosynthetic proteins may be different in different wheat strains [1]. In the present study, our results showed that the contents of several thylakoid proteins (D1, PsaD, and Lhcb4) were markedly low in diploid wheat. In contrast, the levels of these proteins were high in hexaploid wheat and octoploid Triticale. D1, as one of the two reaction center proteins of PSII, is the primary target of photodamage of PSII and subject to repair during photoinhibition [41]. PsaD is necessary for structural organization and function of the PSI complex by interaction with other proteins [42]. Lhcb4 (CP29) is critical to the efficient light harvesting, PSII organization, energy dissipation, and photoprotection in higher plants [43]. Therefore, high levels of D1, PsaD, and Lhcb4 probably suggest that hexaploid wheat and octoploid Triticale have higher photosynthetic capacity than diploid and tetraploid wheat.

## 4. Materials and Methods

### 4.1. Plant Material and Experimental Conditions

Three representative wheat accessions and one genotypes of *Triticale*, diploid accessions (*T. monococcum* L., Z16 with AA genome), tetraploid accessions (*T. dicoccum* Schuebi., H89 with AABB genome), hexaploid accessions (*T. aestivum* L., CN19 with AABBDD genome) and octaploid *Triticale* accessions (XZ31 with AABBDDRR genome) were used in this experiment (Table 3). The seeds were provided by Sichuan Agricultural University, Ya’an, China.

Experiments were carried out at Sichuan Agricultural University in Ya’an (29°59′ N, 103°00′ E), Sichuan Province, China. The tested seeds of all genotypes were selected and sterilized for 10 min with 0.1% NaClO. Then they were thoroughly washed with distilled water three times, and germinated in Petri-dishes at room temperature for two days in the darkness. The germinated seedlings with uniform size were transferred in sterilized sand with 1/2 Hoagland’s solution in a greenhouse (illumination of 250 μmol∙m^−2^∙s^−1^; 75–85% relative humidity; 25/20 °C day/night temperature) until the trefoil stage. For calculating 1000-grain weight, the field experiments of wheat were conducted at the experimental farm of Sichuan Agricultural University in 2016 and 2017 according to the method of Chen et al. [1].

### 4.2. Seed Germination, Seedling Growth, 1000-Grain Weight, and Stomata

Germination rate was determined when 70% of control roots were about 5 mm long [44]. During trefoil stage, seedling growth including root length, the number of roots, height, total length, and leaf area of the second leaves, was measured according to the method of Xiong et al. [45]. Z16, H89, CN19, and XZ31 were harvested manually at full maturity and threshed. Then, 1000 grain-weight was obtained.

The stomatal structure in second leaves was photographed by fluorescence microscopy (Bx53 System, Olympus Corporation, Tokyo, Japan) according to the method of Kondo et al. [46]. The number of stomata in each photograph was calculated and converted into a stomata density. We also measured single stomata perimeter and area.

### 4.3. Pigment Contents, Osmotic Regulators, and Total Protein Content

The contents of chlorophyll (Chl) *a* and *b* were determined by taking fresh seedling leaves (0.5 g) as the previous method described [47]. After filtering, absorbance of the filtrate was recorded at 663 and 645 nm using a UV-visible spectrophotometer (Hitachi-U2000, Tokyo, Japan). Carotenoid were extracted from the fresh leaves with 80% (*v*/*v*) acetone and measured at 480 and 510 nm spectrophotometrically by the previous method [48]. Soluble sugar was extracted from the leaves using an 80% ethanol solution in boiling water. Then its content was colorimetrically measured after reacting with anthrone reagent according to the method of Thomas [49]. Proline were extracted in 3% (*w*/*v*) sulfosalicylic acid and the measurement of its content was performed at 520 nm using a UV spectrophotometer as described previously by Bates et al. [50]. A UV spectrophotometer was applied for the determination of total soluble proteins in fresh leaves (0.5 g), which were homogenized with 5 mL sodium phosphate buffer (pH 7.2) and then centrifuged for 10 min at 4 °C [51].

### 4.4. Measurements of Leaf Chlorophyll Fluorescence and Gas Exchange

Imaging PAM M-Series Chlorophyll Fluorescence System (Heinz-Walz Instruments, Effeltrich, Germany) was applied for measurement of modulated chlorophyll fluorescence according to the user manual. The leaves were dark adapted for 1 h before taking images and reading. Actinic light intensity was set to an irradiance of 180 μmol∙m^−2^∙s^−1^, and the saturated pulse intensity was set to 8000 μmol∙m^−2^∙s^−1^. The maximum efficiency of PSII photochemistry (*Fv*/*Fm*), the photochemical quenching (qP), the quantum yield of PSII electron transport (ΦPSII), the fraction of PSII centers that are open (qL), and the non-photochemical quenching (NPQ) were calculated according to the method of Maxwell and Johnson [52]. The representative image data obtained from each experiment were normalized to a false color scale.

The state of PSI photochemistry and Chl *a* fluorescence in seedling leaves were measured with the dual PAM-100 fluorometer (Heinz-Walz Instruments, Effeltrich, Germany) with a ChlF unit and P700 dual wavelength (830/875 nm) unit according to Klughammer and Schreiber [53]. Plants were kept in the dark for 30 min and for 2 min in the measuring head prior to measurements. The effective quantum yield of PSI (ΦPSI), oxidation status of PSI donor side (Φ_ND_), and reduction status of PSI acceptor side (Φ_NA_) were obtained according to Klughammer and Schreiber [53].

State transition measurement was conducted using whole leaves following Pietrzykowska et al. [54]. Preferential PSII excitation was obtained by a blue-light (40 μmol∙m^−^^2^∙s^−1^) provided by a SL 3500-R-D lamp equipped with a 650-nm interference filter. The seedlings were exposed to red and far-red light treatment with customized LED light sources (SL 3500-R-D) as previously described by Leoni et al. [55]. Fm level in State I (Fm’) and State II (Fm’’) were obtained at the end of each state transition cycle by the application of the saturating light pulse.

Net photosynthetic rate (*P*n), stomatal conductance (Gs), transpiration rate (Tr) and intercellular CO_2_ concentration (Ci) were measured using the GSF-3000 photosynthesis system (Heinz-Walz Instruments, Effeltrich, Germany) at a photosynthetically active radiation (PAR) of 1500 μmol∙photons∙m^−2^∙s^−1^. The 360 μmol∙mol^−1^ CO_2_ concentration and 70% relative humidity at room temperature was used for measurement of the CO_2_ assimilation rate [56].

### 4.5. Measurement of Reactive Oxygen Species (ROS)

Hydrogen peroxide (H_2_O_2_) and superoxide (O_2_^•–^) in seedling leaves were visually recorded with 3,3-diaminobenzidine (DAB) and nitro blue tetrazolium (NBT), respectively. The second leaves were cut at the leaf base and immersed in 2 mg∙mL^−1^ DAB or 0.5 mg∙mL^−1^ NBT solution for 2–8 h with vacuum infiltration in darkness, and then the stained leaves were decolorized in 95% ethanol for 0.5–2 h in a boiling water bath [57]. For further quantitative analysis of ROS, the content of H_2_O_2_ and O_2_^•–^ in leaves was measured according to the previous methods of Okuda et al. [58] and Elstner and Heupel [59], respectively.

### 4.6. Measurement of Antioxidative Enzymes and Non-Enzymatic Antioxidants

For measurements of antioxidant enzymes, the leaves (0.5 g fresh weight) were ground with 5 mL ice-cold 50 mM potassium phosphate buffer (pH 7.8), which contains 2 mM ascorbate, 0.2 mM EDTA and 2% (*w*/*v*) polyvinyl polypyrrolidone (PPVP). After centrifugation (12,000× *g* for 30 min at 4 °C), the supernatants were used for enzyme activity assays. Peroxidase (POD), glutathione peroxidase (GPX), glutathione reductase (GR), ascorbate peroxidase (APX), catalase (CAT), and Superoxide dismutase (SOD), activities were measured according to the previous methods [60].

The contents of reduced ascorbic acid (ASA) and dehydroascorbate (DHA) were measured by an HPLC method following Xu et al. [60]. The extraction and detection to oxidized glutathione (GSSG) and reduced glutathione (GSH) were carried out following Bechtold et al. [61].

### 4.7. Immunoblot Analysis

Based on the method of Chen et al. [62], thylakoid membrane proteins from seedling leaves were isolated under dim light. Then, Chl concentration of the thylakoid preparation was determined as described previously by Porra et al. [47]. The thylakoid proteins were separated by 15% SDS-PAGE, and subsequently shifted to a polyvinyldifluoride (PVDF) membrane (Immobilone, Millipore, Darmstadt, Germany). D1, D2, CP43, Lhcb1, Lhcb2, Lhcb3, Lhcb4, Lhcb5, Lhcb6, PsaD, Lhca1, Lhca2, Lhca3, and Lhca4 were immuno-detected with protein-specific antibodies, which were bought from Agrisera (Umea, Sweden). Then, the horseradish peroxidase-conjugated secondary antibody (Agrisera Comp., Umea, Sweden) and a chemiluminescent detection system (ECL, GE Healthcare, Buckinghamshire, UK) were used for detecting the immunoblotting signals. Final quantitative analysis of the protein bands was carried out with the Quantity One software (Bio-Rad Comp. Hercules, CA, USA).

### 4.8. Statistical Analysis

All analyses were performed in four independent replicates and values were shown as mean ± standard deviation (SD). Data for various parameters among wheat with different ploidy levels and octoploid Triticale was statistically analyzed by one-way ANOVA at the level of *p* < 0.05 using Duncan’s multiplication range test by the SPSS Statistics 19.0 software (IBM, Chicago, IL, USA).

## 5. Conclusions and Perspective

In the present study, we found that wheat with different ploidy levels and octoploid Triticale differed in photosynthesis, ROS accumulation, and antioxidant enzymatic systems. Our results indicated that hexaploid wheat and octoploid Triticale show high photosynthetic capacities and have more effective antioxidant defense systems. Here, we propose that hexaploid wheat and octoploid Triticale have developed more effective photosynthetic apparatus and protective mechanisms in the process of plant evolution. Increase of ploidy levels may make these species more competitive, and therefore might be a strategy for the rapid selection and breeding of excellent wheat varieties under changing agricultural and natural ecosystems. Further research is needed in order to elucidate which genome is more effective in photosynthesis under natural conditions (photoinhibition and high temperature stress usually happen at noon). Also, investigations are necessary to identify which genome has better ROS-scavenging systems under environmental changes.

So far, our results cannot distinguish between the influences of BB genome and tetraploid chromosome structure in the determination of the better performance of tetraploid H89 than diploid Z16. Genomes and genes involved should be independently identified and potential synergistic effects should be further investigated in the future.

## Figures and Tables

**Table 1:** Seed germination, seedlings growth at trefoil stage, and 1000-grain weight (g) in Z16, H89, CN19, and XZ31.

Parameters	Z16	H89	CN19	XZ31
(a) Seed germination				
Germination rate (%)	49.3 ± 0.7 ^c^	62.5 ± 0.6 ^b^	87.8 ± 0.9 ^a^	35.2 ± 1.1 ^d^
Germination potential (%)	39.1 ± 0.3 ^c^	59.2 ± 0.8 ^b^	83.4 ± 0.6 ^a^	32.5 ± 0.5 ^d^
Germination index (%)	31.1 ± 0.5 ^c^	49.3 ± 0.3 ^b^	74.7 ± 0.5 ^a^	27.7 ± 0.6 ^d^
(b) Seedling growth at trefoil stage				
Root length (cm)	8.2 ± 0.3 ^d^	14.5 ± 0.2 ^c^	18.2 ± 0.1 ^b^	19.7 ± 0.3 ^a^
Root number	5.1 ± 0.6 ^b^	5.6 ± 0.3 ^b^	5.4 ± 0.2 ^b^	7.2 ± 0.2 ^a^
Seedling height (cm)	3.3 ± 0.3 ^d^	7.4 ± 0.1 ^a^	4.4 ± 0.2 ^c^	6.3 ± 0.1 ^b^
Total length of seedlings (cm)	25.3 ± 0.3 ^c^	44.2 ± 0.7 ^b^	44.4 ± 0.5 ^b^	46.9 ± 0.5 ^a^
Leaf area (cm^2^)	2.1 ± 0.1 ^d^	4.8 ± 0.1 ^c^	8.7 ± 0.2 ^b^	7.3 ± 0.2 ^a^
(c) 1000-grain weight (g)	7.9 ± 0.1 ^d^	41.4 ± 0.7 ^b^	52.8 ± 0.3 ^a^	33.2 ± 0.3 ^c^

The values are means ± SD from four independent biological replicates. Small letters indicate a significant difference among Z16, H89, CN19, and XZ31 at *p* < 0.05 (Duncan’s multiplication range test). Z16, H89, CN19, and XZ31 present accessions of *Triticum monococcum* (W2n), *Triticum dicoccum* (W4n), *Triticum aestivum* (W6n), and octaploid Triticale (T8n), respectively.

**Table 2:** Stomata of the second leaf and osmotic regulators in Z16, H89, CN19, and XZ31.

Parameters	Z16	H89	CN19	XZ31
(a) Stomata of the second leaves				
Single stomatal perimeter (μm)	135.8 ± 13.8 ^c^	170.3 ± 5.2 ^b^	205.2 ± 6.4 ^b^	232.4 ± 12.7 ^a^
Single stomatal area (μm^2^)	1174 ± 45 ^d^	2067 ± 38 ^c^	2365 ± 55 ^b^	2805 ± 68 ^a^
Stomatal density (per mm^−2^ × 10^−3^)	49.9 ± 2.2 ^a^	29.5 ± 1.6 ^b^	26.9 ± 1.2 ^bc^	24.3 ± 0.8 ^c^
(b) Osmotic regulators				
Soluble sugar content (mg g^−1^ FW)	83.1 ± 0.8 ^d^	90.6 ± 0.9 ^c^	115.8 ± 1.0 ^b^	130.3 ± 0.6 ^a^
Proline content (mg g^−1^ FW)	37.8 ± 0.8 ^b^	41.2 ± 1.6 ^b^	51.6 ± 2.4 ^a^	51.2 ± 2.0 ^a^
(c) Total protein content (mg g^−1^ FW)	7.8 ± 0.1 ^b^	12.6 ± 0.2 ^a^	13.3 ± 0.4 ^a^	12.2 ± 0.1 ^a^

The values are means ± SD from four independent biological replicates. Small letters indicate a significant difference among Z16, H89, CN19, and XZ31 at *p* < 0.05 according to Duncan’s multiplication range test. Z16, H89, CN19, and XZ31 present accessions of *Triticum monococcum* (W2n), *Triticum dicoccum* (W4n), *Triticum aestivum* (W6n), and octaploid Triticale (T8n), respectively.

**Table 3:** Details of species, accession, source, ploidy level, genome, ear and grain characteristics, and origin of genetic materials used in the study.

Species	Accession	Genome	Sources	Ploidy	Ear	Grain
*T. monococcum*	Z16 (W2n)	AA	China	Diploid (2n = 2x = 14)	Dehiscent	Hulled
*T. dicoccum*	H89 (W4n)	AABB	China	Tetraploid (2n = 4x = 28)	Indehiscent	Naked
*T. aestivum*	CN19 (W6n)	AABBDD	China	Hexaploid (2n = 6x = 42)	Indehiscent	Naked
*Triticale*	XZ31 (T8n)	AABBDDRR	China	Octaploid (2n = 8x = 56)	Indehiscent	Naked

*T*. = *Triticum*. W = wheat.

## Supplementary Materials

Supplementary materials can be found at http://www.mdpi.com/1422-0067/19/10/3006/s1.

## Abbreviations

PSII	Photosystem II
Chl	Chlorophyll
ROS	Reactive oxygen species
Pn	Net photosynthetic rate
NPQ	Non-photochemical quenching
Fv/Fm	Maximum quantum yield of PSII
ΦPSII	Effective quantum yield of PSII
Qp	Photochemical quenching
Ql	Fraction of PSII centers that are open

## References

[1] Chen Y.E., Su Y.Q., Zhang C.M., Ma J., Mao H.T., Yang Z.H., Yuan M., Zhang Z.W., Yuan S., Zhang H.Y. (2017). Comparison of photosynthetic characteristics and antioxidant systems in different wheat strains. J. Plant Growth Regul..

[2] Wang J.Y., Turner N.C., Liu Y.X., Siddique K.H.M., Xiong Y.C. (2017). Effects of drought stress on morphological, physiological and biochemical characteristics of wheat species differing in ploidy level. Funct. Plant Biol..

[3] Tanno K., Willcox G. (2006). How fast was wild wheat domesticated?. Science.

[4] Gustafson P., Raskina O., Ma X.F., Nevo E., Carver B.F. (2009). Wheat evolution, domestication, and improvement. Wheat Science and Trade.

[5] Brestic M., Zivcak M., Hauptvogel P., Misheva S., Kocheva K., Yang X.H., Li X.N., Allakhverdiev S.I. (2018). Wheat plant selection for high yields entailed improvement of leaf anatomical and biochemical traits including tolerance to non-optimal temperature conditions. Photosynth. Res..

[6] Chantret N., Salse J., Sabot F., Rahman S., Bellec A., Laubin B., Dubois I., Dossat C., Sourdille P., Joudrier P. (2005). Molecular basis of evolutionary events that shaped the *Hardness* locus in diploid and polyploid wheat species (Triticum and Aegilops). Plant Cell.

[7] Dou Q.W., Tanaka H., Nakata N., Tsujimoto H. (2006). Molecular cytogenetic analyses of hexaploid lines spontaneously appearing in octoploid Triticale. Theor. Appl. Genet..

[8] Watanabe N., Kobayashi S., Furuta Y. (1997). Effect of genome and ploidy on photosynthesis of wheat. Euphytica.

[9] Li M.S., Wang C.Y., Song J.Q., Chi Y.G., Wang X.F., Wu Y.F. (2008). Evolutional trends of leaf stomatal and photosynthetic characteristics in wheat evolutions. Acta Ecol. Sin..

[10] Bilgrami S.S., Houshmand S.A., Khodambashi M., Zandi P., Siavoshi M., Khademi S., Navabpour S., Nasiri-Dehbaneh M., Amoue H., Tadayyon M.R. (2015). Photosynthetic performance in ploidy levels and amphyploids of wheat during developmental stages. J. Anim. Plant Sci..

[11] Del Blanco I.A., Rajaram S., Kronstad W.E., Reynolds M.P. (2000). Physiological performance of synthetic hexaploid wheat-derived populations. Crop Sci..

[12] Nygren J., Shad N., Kvarnheden A., Westerbergh A. (2015). Variation in susceptibility to *wheat dwarf virus* among wild and domesticated wheat. PLoS ONE.

[13] Austin R.B., Morgan C.L., Ford M.A., Bhagwat S.G. (1982). Flag leaf photosynthesis of *Triticum aestivum* and related diploid and tetraploid Species. Ann. Bot..

[14] Wang C.Y., Li M.S., Song J.Q., Chi Y.G., Wang X.F., Wu Y.F. (2008). Differences in stomatal and photosynthetic characteristics of five diploidy wheat species. Acta Ecol. Sin..

[15] Zhang S.Q., Shan L. (2003). Difference of water use efficiency of diploidy wheat species with different chromosome set and its relationship with root system growth. Acta Agron. Sin..

[16] Planchon C., Fesquet J. (1982). Effect of the D genome and of selection on photosynthesis in wheat. Theor. Appl. Genet..

[17] Austin R.B., Ford M.A., Miller T.E., Morgan C.L., Parry M.A.J., Biggens J. (1987). Variation in photosynthetic characteristics among Triticum species and attempts to exploit it in breeding. Progress in Photosynthesis Research.

[18] Huang M.L., Deng X.P., Zhou S.L., Zhao Y.Z. (2007). Grain yield and water use efficiency of diploid, tetraploid and hexaploid wheats. Acta Ecol. Sin..

[19] Halloran G.M., Pennell A.L. (1982). Grain size and seedling growth of wheat at different ploidy levels. Ann. Bot..

[20] Hejnák V., Hniličková H., Hnilička F., Andr J. (2016). Gas exchange and *Triticum* sp. with different ploidy in relation to irradiance. Plant Soil Environ..

[21] Schreiber U., Klughammer C., Neubauer C. (1988). Measuring P700 absorbance changes around 830 nm with a new type of pulse modulation system. Z. Naturforsch..

[22] Jensen P.E., Gilpin M., Knoetzel J., Scheller H.V. (2000). The PSI-K subunit of photosystem I is involved in the interaction between light-harvesting complex I and the photosystem I reaction center core. J. Biol. Chem..

[23] Soltis D.E., Soltis P.S. (1993). Molecular data and the dynamic nature of polyploidy. Crit. Rev. Plant Sci..

[24] Otto S.P., Whitton J. (2000). Polyploid incidence and evolution. Annu. Rev. Genet..

[25] Ramsey J. (2011). Polyploidy and ecological adaptation in wild yarrow. Proc. Natl. Acad. Sci. USA.

[26] Pérez-Alfocea F., Larher F. (1995). Sucrose and proline accumulation and sugar efflux in tomato leaf discs affected by NaCl and polyethylene glycol 6000 iso-osmotic stresses. Plant Sci..

[27] Singh T.N., Paleg I.G., Aspinall D. (1973). Stress Metabolism III. Variations in response to water deficit in the barley plant. Aust. J. Biol. Sci..

[28] Monneveux P., Nemmar M. (1986). Contribution to the study of drought resistance in bread wheat (*Triticum aestivum* L.) and durum wheat (*Triticum durum* Desf.): Study of proline accumulation during development. Agronomie.

[29] Sonoike K. (2011). Photoinhibition of photosystem I. Physiol. Plant..

[30] Brestic M., Zivcak M., Kunderlikova K., Allakhverdiev S.I. (2016). High temperature speciically afects the photoprotective responses of chlorophyll *b*-deficient wheat mutant lines. Photosynth. Res..

[31] Miyake C., Miyata M., Shinzaki Y., Tomizawa K. (2005). CO_2_ response of cyclic electron flow around PSI (CEF-PSI) in tobacco leaves–relative electron fluxes through PSI and PSII determine the magnitude of non-photochemical quenching (NPQ) of Chl fluorescence. Plant Cell Physiol..

[32] Ashraf M., Harris P.J.C. (2013). Photosynthesis under stressful environments: An overview. Photosynthetica.

[33] Kalaji H.M., Schansker G., Brestic M., Bussotti F., Calatayud A., Ferroni L., Goltsev V., Guidi L., Jajoo A., Li P. (2017). Frequently asked questions about chlorophyll fluorescence, the sequel. Photosynth. Res..

[34] Graßes T., Pesaresi P., Schiavon F., Varotto C., Salamini F., Jahns P., Leister D. (2002). The role of ∆pH-dependent dissipation of excitation energy in protecting photosystem II against light-induced damage in *Arabidopsis thaliana*. Plant Physiol. Biochem..

[35] Kramer D.M., Johnson G., Kiirats O., Edwards G.E. (2004). New fluorescence parameters for the determination of Q_A_ redox state and excitation energy fluxes. Photosynth. Res..

[36] James R.A., von Caemmerer S., Condon A.G., Zwart A.B., Munns R. (2008). Genetic variation in tolerance to the osmotic stress component of salinity stress in durum wheat. Funct. Plant Biol..

[37] Gill S.S., Tuteja N. (2010). Polyamines and abiotic stress tolerance in plants. Plant Signal. Behav..

[38] Horváth E., Szalai G., Janda T. (2007). Induction of abiotic stress tolerance by salicylic acid signaling. J. Plant Growth Regul..

[39] Noctor G., Foyer C.H. (1998). Ascorbate and glutathione: Keeping active oxygen under control. Annu. Rev. Plant Physiol. Plant Mol. Biol..

[40] Asada K. (1999). The water-water cycle in chloroplasts: Scavenging of active oxygens and dissipation of excess photons. Annu. Rev. Plant Physiol. Plant Mol. Biol..

[41] Nath K., Jajoo A., Poudyal R.S., Timilsina R., Park Y.S., Aro E.M., Nam H.G., Lee C.H. (2013). Towards a critical understanding of the photosystem II repair mechanism and its regulation during stress conditions. FEBS Lett..

[42] Li N., Zhao J.D., Warren P.V., Warden J.T., Bryant D.A., Golbeck J.H. (1991). PsaD is required for the stable binding of PsaC to the photosystem I core protein of *Synechococcus* sp. PCC 6301. Biochemistry.

[43] Chen Y.E., Zhao Z.Y., Zhang H.Y., Zeng X.Y., Yuan S. (2013). The significance of CP29 reversible phosphorylation in thylakoids of higher plants under environmental stresses. J. Exp. Bot..

[44] Munzuroglu O., Geckil H. (2002). Effects of metals on seed germination, root elongation, and coleoptile and hypocotyl growth in *Triticum aestivum* and *Cucumis sativus*. Arch. Environ. Contam. Toxicol..

[45] Xiong Y.C., Li F.M., Zhang T. (2006). Performance of wheat crops with different chromosome ploidy: Root-sourced signals, drought tolerance, and yield performance. Planta.

[46] Kondo T., Kajita R., Miyazaki A., Hokoyama M., Nakamura-Miura T., Mizuno S., Masuda Y., Irie K., Tanaka Y., Takada S. (2010). Stomatal density is controlled by a mesophyll-derived signaling molecule. Plant Cell Physiol..

[47] Porra R.J., Thompson W.A., Kriedemann P.E. (1989). Determination of accurate extinction coefficients and simultaneous equations for assaying chlorophylls *a* and *b* extracted with four different solvents: Verification of the concentration of chlorophyll standards by atomic absorption spectroscopy. Biochim. Biophys. Acta.

[48] Jensen A., Hellebust A., Crargie J.S. (1978). Chlorophylls and carotenoids. Handbook of Phycological Methods: Physiological and Biochemical Methods.

[49] Thomas T.A. (1977). An automated procedure for the determination of soluble carbohydrates in herbage. J. Sci. Food Agric..

[50] Bates L.S., Waldren R.P., Teare I.D. (1973). Rapid determination of free proline for water-stress studies. Plant Soil.

[51] Lowry O.H., Rosebrough N.J., Farr A.L., Randall R.J. (1951). Protein measurement with the Folin phenol reagent. J. Biol. Chem..

[52] Maxwell K., Johnson G.N. (2000). Chlorophyll fluorescence–a practical guide. J. Exp. Bot..

[53] Klughammer C., Schreiber U. (1994). An improved method, using saturating light pulses, for the determination of photosystem I quantum yield via P700^+^-absorbance changes at 830 nm. Planta.

[54] Pietrzykowska M., Suorsa M., Semchonok D.A., Tikkanen M., Boekema E.J., Aro E.M., Jansson S. (2014). The light-harvesting chlorophyll *a/b* binding proteins Lhcb1 and Lhcb2 play complementary roles during state transitions in *Arabidopsis*. Plant Cell.

[55] Leoni C., Pietrzykowska M., Kiss A.Z., Suorsa M., Ceci L.R., Aro E.M., Jansson S. (2013). Very rapid phosphorylation kinetics suggest a unique role for Lhcb2 during state transitions in Arabidopsis. Plant J..

[56] Yamori W., Noguchi K., Hanba Y.T., Terashima I. (2006). Effects of internal conductance on the temperature dependence of the photosynthetic rate in spinach leaves from contrasting growth temperatures. Plant Cell Physiol..

[57] Chen Y.E., Cui J.M., Su Y.Q., Yuan S., Yuan M., Zhang H.Y. (2015). Influence of stripe rust infection on the photosynthetic characteristics and antioxidant system of susceptible and resistant wheat cultivars at the adult plant stage. Front. Plant Sci..

[58] Okuda T., Matsuda Y., Yamanaka A., Sagisaka S. (1991). Abrupt increase in the level of hydrogen peroxide in leaves of winter wheat is caused by cold treatment. Plant Physiol..

[59] Elstner E.F., Heupel A. (1976). Inhibition of nitrite formation from hydroxylammoniumchloride: A simple assay for superoxide dismutase. Anal. Biochem..

[60] Xu J., Zhu Y.Y., Ge Q., Li Y.L., Sun J.H., Zhang Y., Liu X.J. (2012). Comparative physiological responses of *Solanum nigrum* and *Solanum torvum* to cadmium stress. New Phytol..

[61] Bechtold U., Murphy D.J., Mullineaux P.M. (2004). Arabidopsis peptide methionine sulfoxide reductase2 prevents cellular oxidative damage in long nights. Plant Cell.

[62] Chen Y.E., Yuan S., Schröder W.P. (2016). Comparison of methods for extracting thylakoid membranes of *Arabidopsis* plants. Physiol. Plant..

