# Supplementary material for: Comparison on Photosynthesis and Antioxidant Defense Systems in Wheat with Different Ploidy Levels and Octoploid Triticale
Source: Int J Mol Sci. 2018 Oct 2;19(10):3006. doi: 10.3390/ijms19103006 (PMC6213355; doi:10.3390/ijms19103006)
Supplement: Supplementary file 1 [file ijms-19-03006-s001.pdf]

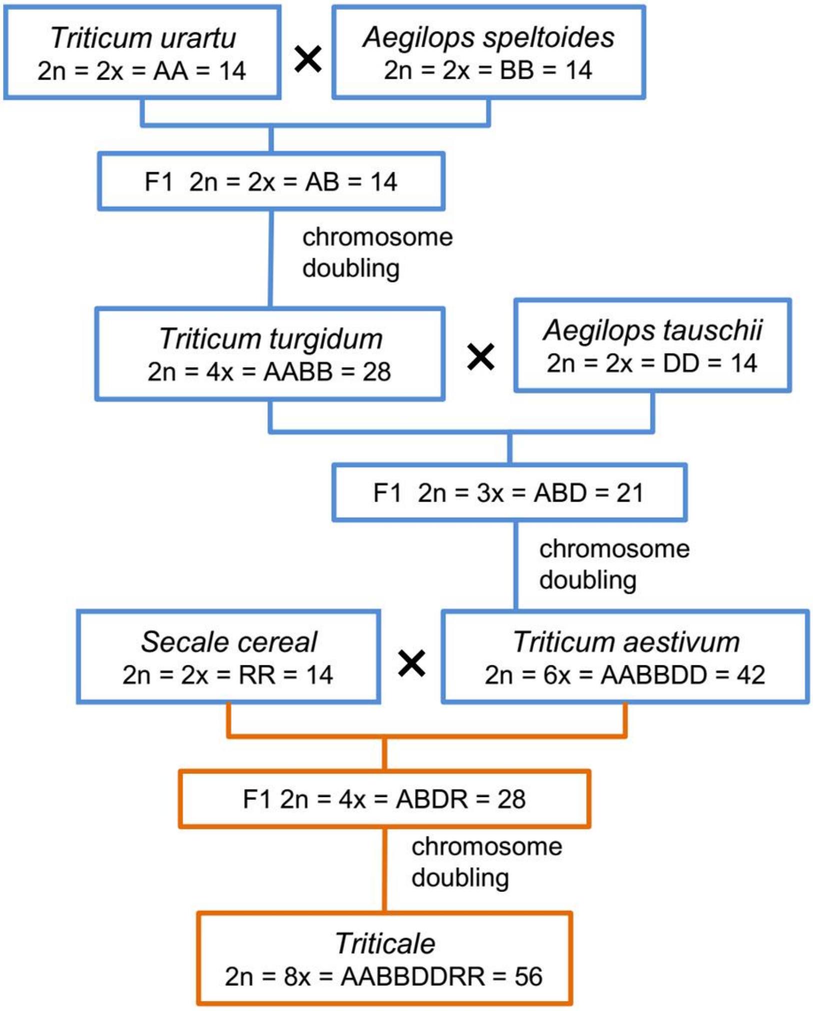

Figure S1. Genetic background of wheat with different ploidy levels and octoploid Triticale during the evolutionary process. Blue and orange indicate natural hybridization and man-made crossing, respectively.

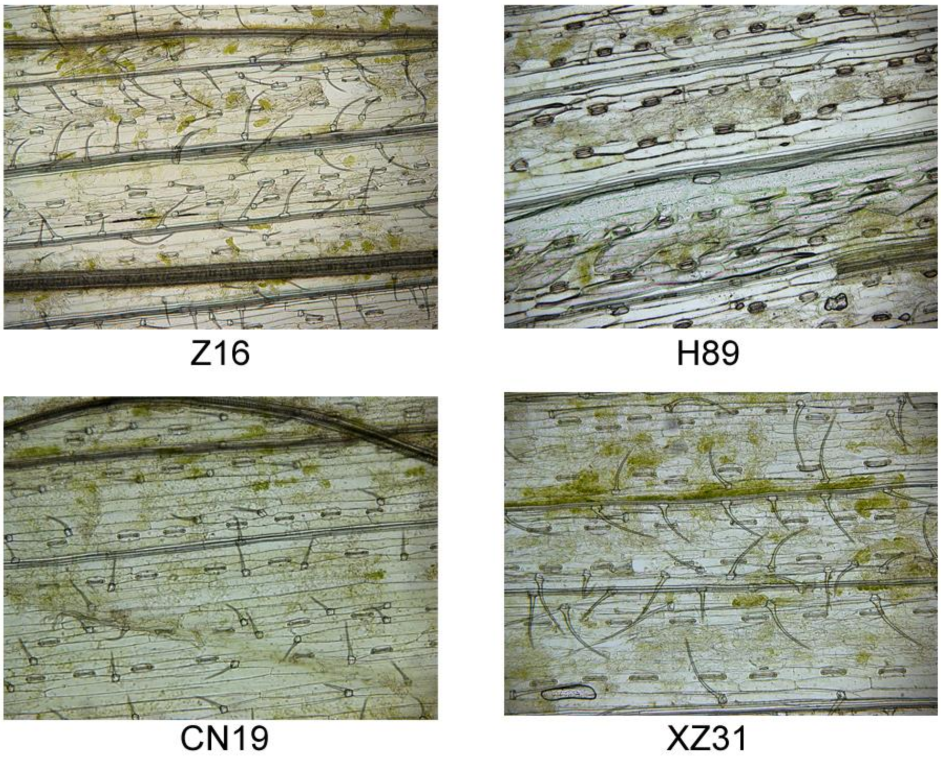

Figure S2. Analysis of stomatal characteristics of wheat with different ploidy levels and octoploid Triticale. Z16, accessions of *Triticum monococcum* (W2n); H89, accessions of *Triticum dicoccum* (W4n); CN19, accessions of *Triticum aestivum* (W6n); XZ31, accessions of octaploid Triticale (T8n).

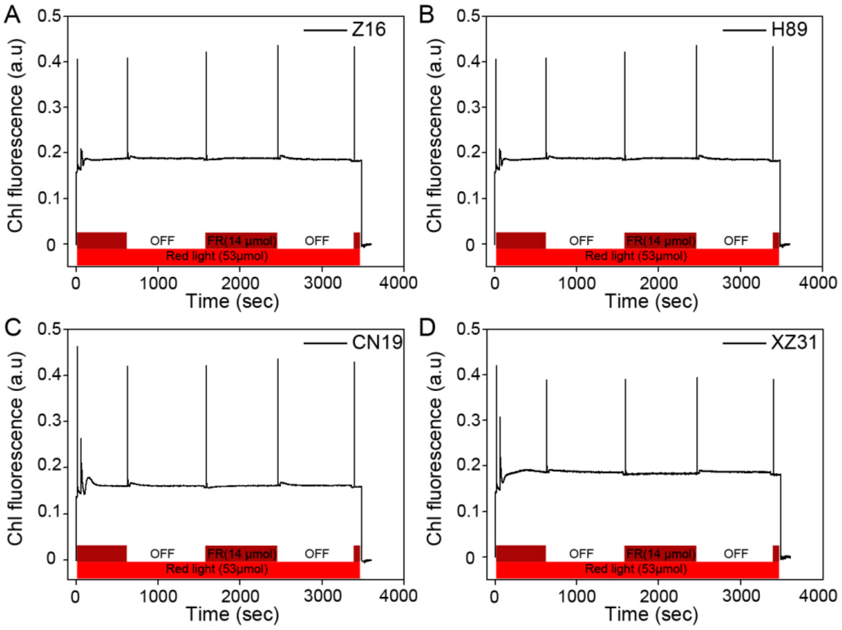

Figure S3. Assays of state transitions in Z16, H89, CN19, and XZ31. Pulse amplitude-modulated fluorescence traces after shifts from state 1 to state 2 light and back for Z16 (**A**), H89 (**B**), CN19 (**C**), and XZ31 (**D**), respectively. The bars at the bottom indicate illumination with red (shown in red) and far-red (dark red) light. Fluorescence is shown in arbitrary units. Z16, H89, CN19, and XZ31 present accessions of *Triticum monococcum* (W2n), *Triticum dicoccum* (W4n), *Triticum aestivum* (W6n), and octaploid Triticale (T8n), respectively.

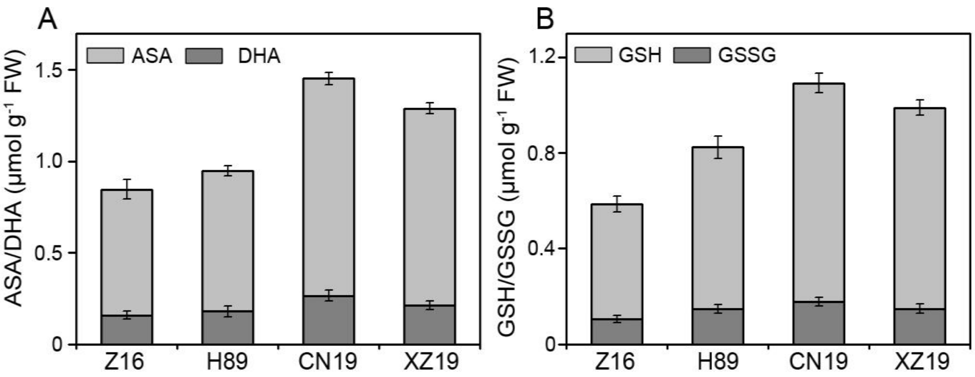

Figure S4. The content of ASA/DHA (A) and GSH/GSSG (**B**) in Z16, H89, CN19, and XZ31. Values are expressed as the means ± SD (n = 4). Z16, H89, CN19, and XZ31 present accessions of *Triticum monococcum* (W2n), *Triticum dicoccum* (W4n), *Triticum aestivum* (W6n), and octaploid Triticale (T8n), respectively.

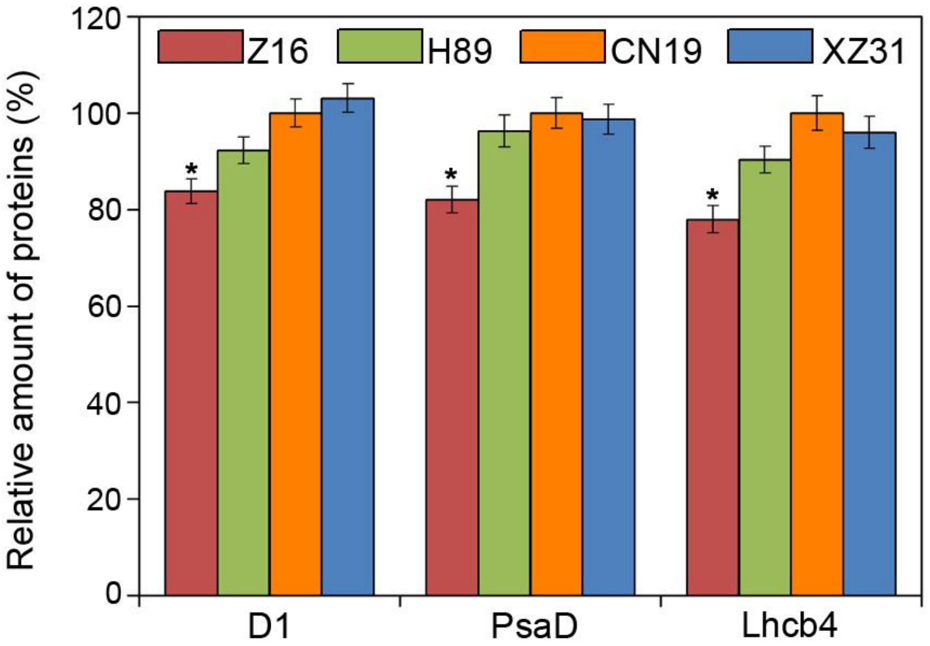

Figure S5. Quantification of D1, PsaD, and Lhcb4 proteins in Z16, H89, CN19, and XZ31. Data are presented relative to the amount of respective CN19 (100%). Values are means ± SD from four independent biological replicates (n = 4). Asterisks indicate statistically significant differences at P < 0.05 level (Duncan's multiplication range test). Z16, accessions of *Triticum monococcum* (W2n); H89, accessions of *Triticum dicoccum* (W4n); CN19, accessions of *Triticum aestivum* (W6n); XZ31, accessions of octaploid Triticale (T8n).

Table S1. Results of two-way analysis of variance (ANOVA) of 1000-grain weight and chlorophyll fluorescence, P700, and gas exchange parameters.

Dependent variable	Pearson Correlation Coefficient	Dependent Variable	Pearson Correlation Coefficient
Fv/Fm	0.597**	Φ_{ND}	0.384
ΦPSII	0.618**	Φ_{NA}	−0.553*
NPQ/4	0.629**	*P*n	0.550*
qP	0.535*	Tr	0.279
*P*m	0.582*	Gs	0.383
qL	−0.725**	Ci	0.414
ΦPSI	0.664**	Chl	0.896**

Numbers represent *F* values at 5% level. *****, ** indicate statistically significant correlation at $P < 0.05$ and $P < 0.01$ levels, respectively. Z16, H89, CN19, and XZ31 present accessions of *Triticum monococcum* (W2n), *Triticum dicoccum* (W4n), *Triticum aestivum* (W6n), and octaploid Triticale (T8n), respectively. Fv/Fm, the maximum efficiency of PSII photochemistry; ΦPSII, the quantum yield of PSII electron transport; NPQ, the non-photochemical quenching; qP, the photochemical quenching; *P*m, maximal P700 signal; qL, the fraction of PSII centers that are open; ΦPSI, effective quantum yield of PSI; Φ_{ND}, quantum yield of non-photochemical energy dissipation in PSI reaction centers due to donor-side limitation; Φ_{NA}, quantum yield of non-photochemical energy dissipation of PSI reaction centers due to acceptor side limitation; *P*n, Net photosynthetic rate; Tr, transpiration rate; Gs, stomatal conductance; Ci, intercellular CO2 concentration; Chl, chlorophyll.
